# Patent challenges in the procurement and supply of generic new essential medicines and lessons from HIV in the southern African development community (SADC) region.

**DOI:** 10.1186/s40545-018-0157-7

**Published:** 2018-12-04

**Authors:** Ellen F. M. ‘t Hoen, Tapiwanashe Kujinga, Pascale Boulet

**Affiliations:** 1 Global Health Unit, University Medical Center Groningen, University of Groningen, Hanzeplein 1, Groningen, GZ 9713 The Netherlands; 2 Medicines Law & Policy, Amsterdam, The Netherlands; 3 Director Pan-African Treatment Access Movement (PATAM), 4th floor Livingstone House, 48 Samora Machel Avenue, Harare, Zimbabwe; 4 Medicines Law & Policy, 105 route de Lossiège, 74130 Contamine sur Arve, France

**Keywords:** HIV, ARVs, Intellectual property, Patents, TRIPS flexibilities, Access to medicines, SADC

## Abstract

High medicines prices increasingly pose challenges for universal access to treatments of communicable and non-communicable diseases. New essential medicines are often patent-protected which sustains high prices in many countries, including in low- and middle-income countries. To respond to the HIV/AIDS crisis of the late nineties and to increase access to antiretroviral treatment, certain flexibilities contained in the Agreement on Trade Related Aspects of Intellectual Property Rights (TRIPS flexibilities) have been clarified and in some respects strengthened at the global level. They have been applied by a number of countries to ensure access to lower-priced generic medicines to treat HIV/AIDS. Governments in the South African Development Community (SADC) have also used TRIPS flexibilities to gain access to lower-priced generic medicines. This paper documents 15 instances of the use of TRIPS flexibilities by eight SADC Member States during the period 2001–2016. Of those, six concerned least developed countries (LDCs) that declared non-enforcement of pharmaceutical patents pursuant to a new LDC transition provision. All instances occurred in the context of medicines procurement for HIV treatment. Such flexibilities can, however, also be used to overcome patent barriers to gain access to generic medicines for other diseases, including NCDs. The SADC, being a regional bloc with over 50% least developed country Members, can make use of the regional exception, a TRIPS flexibility that facilitates the production or procurement of generic medicines to the benefit of the entire region. SADC’s Pharmaceutical Business Plan proposes strategies for increased collaboration and pooled procurement of medicines.

## Background

The Southern African Development Community (SADC) has several legal options that can be used to increase the availability of generic versions of patented medicines and to facilitate regional procurement of medicines. These legal options are known as ‘TRIPS flexibilities’. The aim of this paper is to present data on the application by SADC countries of TRIPS flexibilities between 2001 and 2016 in the context of the response to the HIV/AIDS crisis and to explore their potential future use in the SADC region in efforts to increase access to medicines beyond HIV. The paper describes the legal options of the SADC based on a legal analysis of the relevant provisions of the WTO TRIPS Agreement and the Doha Declaration on the TRIPS Agreement and Public Health.

## Introduction

The Southern African Development Community (SADC) is a regional bloc established in 1992 to foster regional integration and facilitate poverty eradication in southern Africa through economic development and the ensuring of peace and security. As of 2018, it comprises 16 countries: Angola, Botswana, the Comoros, Democratic Republic of the Congo, Lesotho, Madagascar, Malawi, Mauritius, Mozambique, Namibia, Seychelles, South Africa, eSwatini (formerly Swaziland), Tanzania, Zambia, and Zimbabwe [1].

The SADC bears a disproportionate burden of HIV, tuberculosis, and malaria, and has a growing incidence of non-communicable diseases such as heart disease, diabetes, and cancer. South Africa, for example, is home to the largest population of people living with HIV, estimated at 7.1 million as of 2016 [2]. With only 0.7% of the world’s population, the country accounts for 17% of the global HIV population [3]. eSwatini and Botswana have the highest adult HIV prevalence rates globally, at 27.2 and 26.3%, respectively [4, 5].

High pharmaceutical prices driven by patents challenge access to new essential medicines that are needed to treat people suffering from these illnesses. However, the SADC has several legal options that can be used to increase the availability of lower-cost generic medicines. These legal options are known as ‘TRIPS flexibilities’, in reference to the World Trade Organization’s Trade-Related Aspects of Intellectual Property Rights Agreement (TRIPS). TRIPS flexibilities were incorporated in order to safeguard governments’ rights to protect public health and promote access to medicines for all.

This paper describes TRIPS flexibilities most relevant in medicines procurement, documents their use in SADC, and explores their potential future use in the SADC region in efforts to increase access to medicines beyond HIV.

## High medicines prices and the World Trade Organization

Pharmaceutical patents maintain drug prices well above the cost of production and can restrict access to needed medicines. This challenge was made particularly clear during the HIV/AIDS crisis in the late nineties. At that time, it was believed that 40 million people were infected with HIV in the developing world; 24.5 million of them lived in sub-Saharan Africa [6] — and only one in a thousand had access to the antiretroviral medicines (ARVs) used to treat HIV. Over 8000 people died of HIV/AIDS daily in the developing world. [6] In 1996, effective ARVs had become available in high-income countries. However, these ARVs were not, or were only very sparsely, available in low- and middle-income countries, and even then only at very high prices from patent-holding companies. [7]

Conflicts about HIV medicines patents broke out only a few years after the establishment of the World Trade Organization (WTO) and the adoption of the WTO TRIPS Agreement, which set international standards for the protection of intellectual property (IP), including the requirement to provide 20-year patent protection for medicines.

ARV medicines patent protection meant that even when Indian generic companies started to produce generic low-priced ARVs in the late nineties, many people living with HIV in other countries where those products were patented could not gain access to them. One important reason was that procurement agencies such as UNICEF and the International Dispensary Association (IDA) as well as non-governmental organisations (NGOs) such as Médecins sans Frontières (MSF) could not distribute generic ARVs in countries where such medicines were protected by patents [8, 9].

This affected the South African Development Community (SADC) region, in particular since nine^1^ of the SADC members are also members of the African Regional Intellectual Property Office (ARIPO). 1 The regional patent offices of ARIPO and the Organisation Africaine de la Propriété Intellectuelle (OAPI) offer routes to obtain patents in groups of countries and thus provide an easy path for medicines patenting. OAPI grants regional patents automatically effective in all its Member States. ARIPO Member States have the right to reject ARIPO patent applications, in accordance with their own patent law, within 6 months. Countries that tried to improve access to lower-priced ARVs by dealing with the patent situation were confronted with trade retaliation by high-income nations or legal actions by patent-holding companies [10, 11]. This had a chilling effect on governments willingness to purchase and import generic antiretroviral treatment, in spite of the ongoing health emergency.

## Growing recognition regarding the need for TRIPS flexibilities: The Doha declaration on TRIPS and public health

The availability of low-priced generic medicines is a cornerstone of policies designed to secure access to essential medicines. The magnitude of the HIV/AIDS crisis brought the international community together in formulating a response to facilitate access to the low-cost diagnostics, medicines, and other tools needed for prevention, treatment, and care for people living with HIV [12].

In 1999, the World Health Assembly (WHA), the World Health Organization’s (WHO) decision making body, opted to strengthen the role of the WHO in intellectual property ‘to ensure that public health interests are paramount in pharmaceutical and health policies’ [13]. The WHA resolution also urged countries to look into the options they had under international trade rules to safeguard access to essential medicines. Most importantly, the WHA requested the WHO to assess the health implications of international trade agreements such as TRIPS, with a view to assisting countries in mitigating the negative effects of these agreements. The resolution put health advocates at the table of trade negotiations.

In that same year, civil society organisations and treatment activists confronted the WTO Ministerial Conference in Seattle with demonstrations demanding action to introduce greater flexibility into the body of international patent rules to safeguard public health [14]. In April 2001, Boniface Chidyausiku of Zimbabwe who was the chair of the TRIPS Council, the governing body of the TRIPS Agreement, proposed a special session on access to medicines. The chair stressed that the WTO could no longer ignore an issue that was being actively debated outside the WTO but not within it [15]. This proposal led in November 2001 to the adoption of the Declaration on TRIPS and Public Health by the WTO Ministerial Conference in Doha. The so-called ‘Doha Declaration’ confirmed the primacy of public health in intellectual property legislation, clarified certain TRIPS flexibilities, and proposed that two other flexibilities be created [16].

The Doha Declaration was an important policy development in rebalancing the rights of patent holders with the rights and duties of countries to protect public health and in particular to promote access to medicines for all [16]. It recognised the concerns in respect to the effects of IP on high drug prices and provided important political support for the notion that the protection of public health should be paramount in implementing the TRIPS Agreement. It did so in paragraph four of the Declaration, which reads:

*We agree that the TRIPS Agreement does not and should not prevent Members from taking measures to protect public health. Accordingly, while reiterating our commitment to the TRIPS Agreement, we affirm that the Agreement can and should be interpreted and implemented in a manner supportive of WTO Members’ right to protect public health and, in particular, to promote access to medicines for all.*

The Doha Declaration described practical legal tools countries can use to ensure medicines accessibility, known as ‘TRIPS Flexibilities’. Specifically, the Doha Declaration clarified the right to issue compulsory licences, including government use licences. It further clarified the right of countries to adopt international exhaustion regimes so as to permit parallel importation. The Declaration further established a new LDC transition measure specifically with respect to the grant and enforcement of pharmaceutical product patents and test data protection. The Doha Declaration recognised the limitation of the use of compulsory licensing predominantly for export and instructed the Council for TRIPS to find an expeditious solution.

Two TRIPS flexibilities in particular have played and stand to play a critical role in the procurement of lower-priced generic medicines in the SADC: compulsory licensing of patents under TRIPS Article 31 (including public non-commercial use or government use) and the transition provisions for Least Developed Country Members (LDCs) of the WTO. These measures are described in more detail in the following two sections; the flow chart below also details which measures are available to governments depending on their specific circumstances. (Illustration 1)

**Illustration 1:**
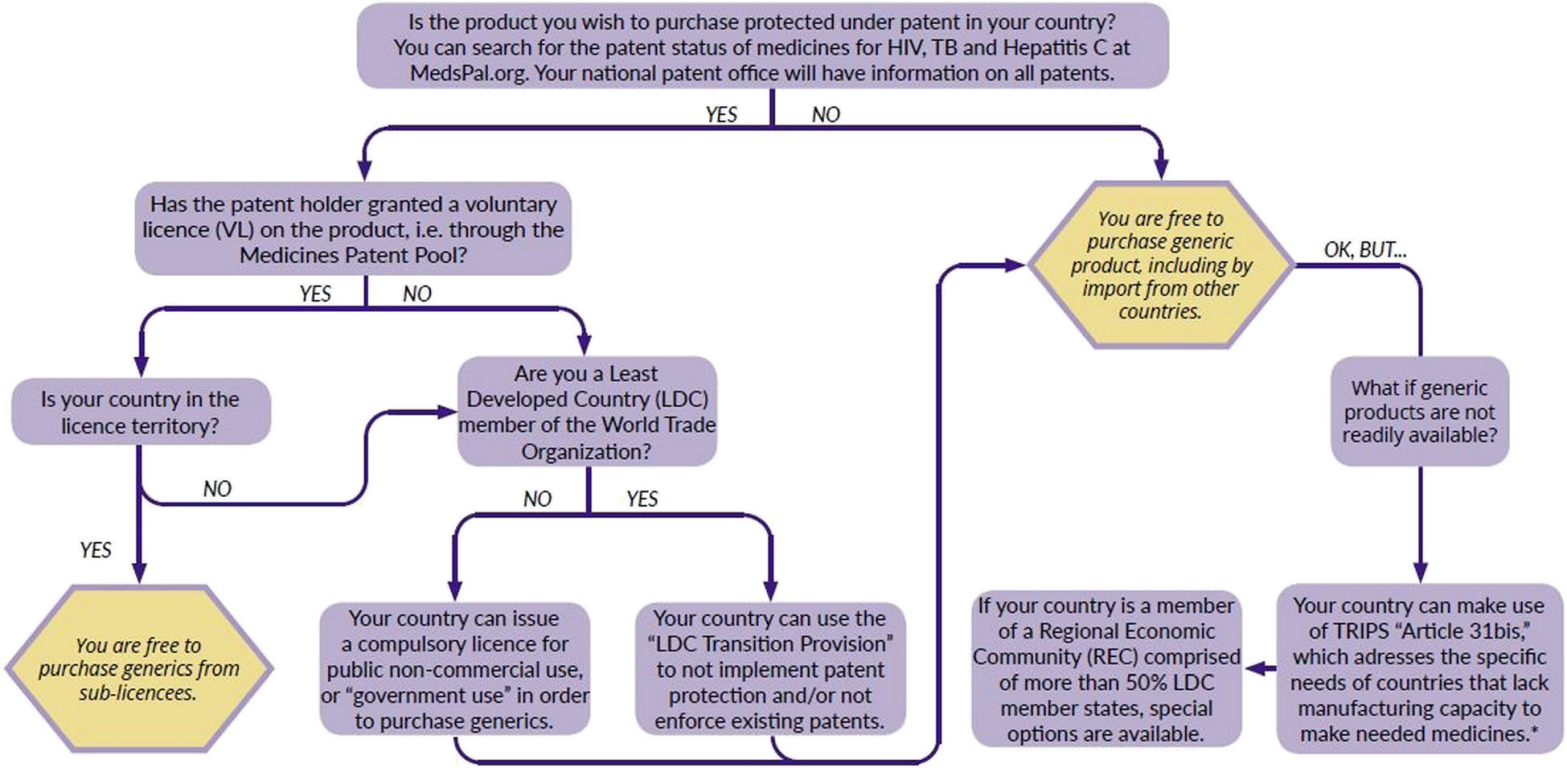
Decision flowchart for determining the application of TRIPS flexibilities in government procurement of medicines [17]

While the need to respond to the HIV treatment crisis drove the change for greater flexibility in IP law, it should be noted that TRIPS flexibilities can be applied for all diseases by all SADC countries.

## Compulsory licensing and public non-commercial use

A compulsory licence allows the use of a patent without the consent of the patent holder against the payment of a reasonable royalty. A compulsory license can be granted following request, for example, from a generic manufacturer who has failed to obtain consent from the patent holder to use the patent. When the government makes use of a patent without the consent of the patent owner for its own purposes this is called ‘public non-commercial use’ (wording of TRIPS Article 31) or ‘government use’. Public non-commercial use can be highly useful in the procurement of medicines for the supply of the health care system when the patent-holding originator company offers the product at an unaffordable price and lower-priced generic versions are available elsewhere or could be produced by local companies. Governments can use compulsory licensing, including government use, to allow for local generic production (of which a part may also be exported as long as the authorised production is predominantly for the supply of the domestic market) as well as import of the medicine needed. If no sources of generic product are available for import and local production is not an option, the TRIPS Agreement also provides for a special compulsory licence predominantly for export that allows another country to manufacture the medicines for sale to the country that needs (but cannot make) them [18]. In paragraph 5(b), the Doha Declaration clarifies that ‘Each Member has the right to grant compulsory licences and the freedom to determine the grounds upon which such licences are granted’ [16].

## Transition provisions for least developed countries

LDC Members of the WTO enjoy the greatest degree of flexibility with regards to pharmaceutical intellectual property. These are called ‘transition provisions’ as they are part of a package that gives LDC Members of the WTO the right to postpone the implementation of certain intellectual property protection provisions. Paragraph 7 of the Doha Declaration specifically removes the obligation for LDCs to comply with Section 5 (Patents) and Section 7 (Protection of Undisclosed Information) of Part II of the TRIPS Agreement, including any obligation to enforce rights under these provisions [19]. This provides LDCs the greatest flexibility with regards to medicines patents and can assist where patents have already been granted.

These flexibilities are important, as some medicines can be widely patented throughout the developing world, including in low-income countries and LDCs [9]. 12 of the 17 OAPI members and 12 of the 18 ARIPO members are WTO LDC Members^2^,^3^. Therefore, the ability of LDCs to not enforce medicines patents remains of key importance. To provide much needed legal certainty for suppliers and procurement agencies^4^ – including non-profit organisations – that seek to avoid the risk of patent infringement suits, certain LDCs have issued statements that they will not enforce patents referring to the TRIPS LDC transition provision. Today the concern about legal repercussions of the supply of generic medicines in LDCs is less relevant for antiviral medicines for the treatment of HIV and HCV, because LDCs are included in the territory of all Medicines Patent Pool licences for HIV and HCV treatment and those granted by companies directly [20]. However, while the Medicines Patent Pool’s mandate was expanded to include all essential medicines in May 2018, it does not currently hold licences for medicines to treat other diseases that affect people in LDCs, such as non-communicable diseases [21].

The LDC transitional provisions of TRIPS can also facilitate medicines manufacturing, as some LDCs have important pharmaceutical production capacity. Bangladesh, for example, was the first source of generic sofosbuvir, a direct-acting antiviral needed for the treatment of hepatitis C [22]. The LDC transitional provisions offer the possibility to further develop manufacturing capacity in LDCs, including in the SADC region and the African Union, which has significant production capacity [23, 26]. The existence of the LDC transition provision does not preclude the use of compulsory licensing or government use of patents by LDCs should this be necessary because of domestic legal requirements. However, it is noteworthy that the widespread non-enforcement of medicines patents with reference to the LDC transition measure has remained unchallenged [24].

## TRIPS flexibilities in the SADC region

If one or several SADC Member States need to access a medicine that is either too expensive or unavailable in the country or region, they have the right under the TRIPS Agreement, as reaffirmed by the Doha Declaration, to take necessary ‘measures to protect public health’ and to ensure access to such medicines using the above-described flexibilities [26]. In addition, SADC Member States can benefit of the regional exception of TRIPS Article 31bis, a specific flexibility available to some regional groups, which facilitates trade in generic medicines within such groups to harness economies of scale [25].

Depending on the patent status of the medicine in each country, whether SADC Member States are classified as LDCs, and/or whether medicine production capacity exists in the SADC region, various options are available to SADC Member States.

There are three types of situations where SADC members may make use of TRIPS compulsory licensing for public health purposes:

1. If there is capacity to produce a generic version of a patented medicine in the SADC, a compulsory or government use licence for local production under TRIPS Article 31 combined with the regional exception of TRIPS Article 31bis, paragraph 3, [27] enables *supply to all SADC Member States,* to countries where no existing patent would be infringed, or (where needed) a compulsory licence has been issued. To make use of this exception in order to supply a medicine to other SADC Members that share the same public health problem, the SADC country would have to have implemented this TRIPS flexibility in its national law, i.e. that the requirement that compulsory licences have to be ‘predominantly for the supply of the domestic market’ does not apply to export to SADC countries^5^.

2. If there is no capacity to produce a generic version of the medicine in the SADC but the medicine is available from affordable generic source(s) outside the region, a SADC country can issue a compulsory license to import such generics in accordance with TRIPS Article 31, *and re-export such generics to other SADC Member States* as allowed by the regional exception of TRIPS Article 31bis (3). An ordinary compulsory or government use licence, in accordance with TRIPS Article 31, may be required in each country where the generic is re-exported, depending on the patent status of the medicine in each country.

3. If there is no capacity to produce a generic version of the medicine in the SADC, and the medicine does not seem to be available from affordable generic source(s) outside SADC, a compulsory or government use licence based on TRIPS Article 31bis may be required, in addition to a notification to the WTO, to trigger production in a country with such capacity, import into the SADC, and *re-export within the SADC*.

## Instances of the use of TRIPS flexibilities in procurement in the SADC region

After the adoption of the Doha Declaration, many countries used TRIPS flexibilities in the procurement of medicines. A study documenting 176 possible instances of the use of TRIPS flexibilities between 2001 and 2016, by 89 countries showed that 100 (56%) involved compulsory licensing or government use of patents and 40 (22.7%) involved the use of the LDC transition period [24]. Eight of the 15 SADC countries, of which six were LDCs, have made use of a TRIPS flexibility, amounting to 15 instances in total in the region (see Table 1). Of those, six were compulsory licences or government use and nine concerned non-enforcement of patents using the LDC transition provision for pharmaceuticals (paragraph 7 of the Doha Declaration). All instances concerned the provision of medications for the treatment of HIV, though four of the LDCs declared to invoke the measure for all medicines. All cases, except for one (South Africa), were executed. The South African instance concerned the Hazel Tau case [28], in which the South African Competition Commission found two drug companies (GlaxoSmithKline and Boehringer Ingelheim) had abused their dominant positions in the ARV markets, specifically by 1) denying a competitor access to an essential facility, 2) excessive pricing, and 3) engaging in an exclusionary act. As a result, the Competition Commission requested that the Competition Tribunal order compulsory licences to enable the exploitation of the relevant patents to allow the marketing of generic versions of the ARVs concerned, including fixed-dose combinations, in exchange for the payment of a reasonable royalty [29]. Before referral and prosecution of the case by the Competition Tribunal, the companies negotiated a settlement that included voluntary licences for supply of the relevant ARVs in sub-Saharan Africa, thereby avoiding the compulsory licences [30].

**Table 1:** Instances of use of TRIPS flexibilities by SADC countries 2001–2016

Country	Year	WTO Class	Type of flex	Product(s)	Disease	Royalty	Executed
Angola	2005	LDC	Par7	All meds	All	NA	Y
Lesotho	2004	LDC	Par7	ARVs	HIV/AIDS	NA	Y
Lesotho	2006	LDC	Par7	All meds	All	NA	Y
Malawi	2004	LDC	Par7	All meds	All	NA	Y
Malawi	2005	LDC	Par7	ARVs	HIV/AIDS	NA	Y
Mozambique	2005	LDC	Par7	ARVs	HIV/AIDS	NA	Y
South Africa	2003	DC	CL	AZT, 3TC, AZT/3TC, NVP	HIV/AIDS	up to 5%	N
Tanzania	2008	LDC	Par7	All Meds	All	NA	Y
Zambia	2004	LDC	CL	3TC/D4T/NVP	HIV/AIDS	2.5%	Y
Zambia	2004	LDC	Par7	ARVs +	HIV/AIDS +	NA	Y
Zambia	2006	LDC	Par7	ARVs +	HIV/AIDS +	NA	Y
Zimbabwe	2004	DC	CL	ARVs	HIV/AIDS	NK	Y
Zimbabwe	2003	DC	CL	ARVs +	HIV/AIDS +	NK	Y
Zimbabwe	2002	DC	CL	ARVs +	HIV/AIDS +	NK	Y
Zimbabwe	2005	DC	CL	ARVs +	HIV/AIDS	NK	Y

Par 7 = the LDC transition provision, *CL* compulsory licence, *ARVs* antiretroviral medicines, *ARV*+ antiretroviral medicines and other medicines needed in the treatment of HIV/AIDS, *NA* not applicable, *NK* not known

In 2010, UNITAID established the Medicines Patent Pool to ensure the availability of generic HIV medicines by making patent licences available to generic producers. The MPP licenses today include all WHO recommended treatments for HIV, and all sub-Saharan African countries are part of the licences territory. This makes the use of TRIPS flexibilities for HIV medicines in the SADC region redundant. However, such a licensing practice does not yet exist for most other diseases and it remains important to ensure that TRIPS flexibilities can be invoked when needed for reasons of public health.

## SADC and the future use of TRIPS flexibilities

For TRIPS flexibilities to be effective, governments must take action and where needed adopt them in national law in a manner they can be easily invoked. The need to utilise the TRIPS flexibilities in the SADC regional bloc was identified by the Member States as one of the strategies in the regional response against diseases.

In the SADC Pharmaceutical Business Plan 2007–2013 [31], the regional bloc proposed to ‘coordinate the implementation of TRIPS flexibilities to improve access to essential medicines within the SADC region’ and outlined strategies to ensure optimal access to medicines within the region.

Further, in November 2012, the SADC approved the SADC Strategy for Pooled Procurement of Essential Medicines and Health Commodities [32], which proposed the pooled procurement of essential medicines for the SADC using the group-contracting model, delivered incrementally through a staged approach starting with coordinated information exchange and work sharing. It was anticipated that pooled procurement of essential medicines and health commodities would, among other benefits, result in significant financial savings and create reserves for further procurement.

The successor – the Pharmaceutical Business Plan 2015–2019 [33] –further proposed, among other actions, to:

- Collaborate with development partners to enable countries to protect, include and take advantage of the flexibilities that exist in the TRIPS Agreement as well as to assist countries in bilateral trade negotiations to conclude agreements that are not detrimental to public health;
- Strengthen the capacity of Intellectual Property (IP) officers, procurement agencies and Medicines Regulatory agencies on issues of intellectual property and public health; and
- Utilise paragraph 6 system (Doha Declaration) or article 31 bis of the TRIPS Agreement to facilitate local production for export; or importation for re-exportation within SADC as a regional bloc.

A logical next step in the utilisation of the TRIPS flexibilities as contemplated by the Business Plans is the full incorporation of the flexibilities in national legislation.

## Conclusion

Evidence from the scale up of HIV treatment and the procurement of HIV medications in the SADC region shows that the TRIPS flexibilities have been effectively used to procure and supply low-priced generic medicines for the treatment of HIV, at a time when voluntary licenses were not yet available.

TRIPS flexibilities may also be needed for the procurement of lower-priced treatments for other diseases, including non-communicable diseases such as cancer, when patented medicines are too costly. LDCs benefit from maximum flexibility because they are not obliged to grant nor enforce pharmaceutical patents until 2033 and as further extended in the future or until the LDC ceases to be an LDC. SADC, being a regional bloc with over 50% LDC members, can benefit from additional options when it coordinates procurement of medicines at the regional level and uses TRIPS flexibilities when needed. Such coordination is foreseen in the SADC Pharmaceutical Business Plans and the strategy for pooled procurement. It will therefore be important that the TRIPS flexibilities are codified in national law in a manner that makes them easy to invoke in a regional approach to the procurement of medical products. There is a key role for UN agencies to ensure that appropriate health-oriented technical advice and assistance is available to assist the process of legislative change. Additionally, wealthy nations should refrain from explicit or implicit threats, tactics or strategies that undermine the right of WTO Members to use TRIPS flexibilities [34].

## Abbreviations

ARVs: Antiretroviral medicines
IDA: International Dispensary Association
IP: Intellectual property
LDC: Least Developed Country
MSF: Médecins sans Frontières
NGO: Non-governmental organisation
SADC: Southern African Development Community
TRIPS: Agreement on Trade Related Aspects of Intellectual Property Rights
WH: World Health Assembly
WHO: World Health Organization
WTO: World Trade Organization

## Footnotes

1 The countries are: Botswana, Lesotho, Malawi, Mozambique, Namibia, Swaziland, Tanzania, Zambia, Zimbabwe.

2 Benin, Burkina Faso, Central African Republic, Chad, Comoros, Guinea, Guinea-Bissau, Mali, Mauritania, Niger, Senegal and Togo. Organisation Africaine de la Propriété Intellectuelle. Etats Membres. http://www.oapi.int/index.php/fr/oapi/etats-membres. Accessed 12 Jul 2018.

3 Gambia, Lesotho, Liberia, Malawi, Mozambique, Rwanda, Sao Tome and Principe, Sierra Leone, Sudan, Uganda, United Republic of Tanzania and Zambia. African Regional Intellectual Property Organization. About Us. http://www.aripo.org/about-aripo/legal-framework. Accessed 12 Jul 2018.

4 See, for example, a letter from the International Dispensary Association (IDA) in support of these provisions: Kempton K. IDA Foundation letter of support for LDC waiver extension. 2015. http://lists.keionline.org/pipermail/ip-health_lists.keionline.org/2015-March/004956.html. Accessed 25 Jul 2018.

5 See for instance, Botswana Industrial Property Act, Act No. 8 of 2010).
